# Reply: The value of sperm DNA fragmentation testing in clinical practice: routine or case-directed?

**DOI:** 10.1093/hropen/hoag088

**Published:** 2026-10-09

**Authors:** Srisailesh Vitthala, Tarek El-Toukhy, Abha Maheshwari

**Affiliations:** MMC IVF Dubai, Dubai Healthcare City, Dubai, UAE; Aberdeen Centre for Reproductive Medicine (ACRM), University of Aberdeen, Aberdeen, UK; Assisted Conception Unit, Guy’s and St Thomas’ Hospital NHS Trust, London, UK; Aberdeen Fertility Centre, NHS Grampian, Foresterhill, Aberdeen, UK

Dear Editor,

We thank Majzoub and Humaidan (2026) for their thoughtful engagement with our recently published *Human Reproduction Open* Opinion article (Vitthala *et al*., 2026a). Their letter raises important points that allow us to clarify and, where appropriate, refine our position.

First, the screening framework: Majzoub and Humaidan (2026) suggest that evaluating sperm DNA fragmentation (SDF) against Wilson–Jungner criteria is conceptually misapplied (Wilson and Jungner, 1968), as SDF has not been advocated as a population-level screening tool. We agree, and this was precisely our point. Our article (Vitthala *et al*., 2026a) argued that SDF testing is utilized indiscriminately across all infertile men and couples presenting to fertility clinics, which functionally resembles population screening in that context. The Wilson–Jungner framework was used not because it perfectly fits a biomarker, but to demonstrate, using an established evaluative structure, that SDF does not meet the basic performance requirements justifying routine deployment. This is a recognized approach in clinical test appraisal (Andermann *et al*., 2008).)

Majzoub and Humaidan (2026) also note that the Wilson–Jungner criteria are condition-centered, and that SDF is a biomarker rather than a disease entity. We accept this distinction, which is why our article (Vitthala *et al*., 2026a) simultaneously applied diagnostic (STARD) and prognostic frameworks to evaluate SDF on its own terms. The limitations of Wilson–Jungner when applied to biomarkers are well-recognized (Khoury *et al*., 2013), and this reinforces rather than undermines our cautionary conclusion.

Second, we acknowledge that the checklist format used in Tables 1, 2, and 4 involves clinical judgment and that some assessments are context-dependent. This is inherent to any structured appraisal of a test with heterogeneous evidence. The frameworks we applied Wilson–Jungner, STARD (Bossuyt *et al*., 2003), Fryback and Thornbury (1991), and the prognostic model evaluation criteria (Altman and Royston, 2000; Vickers and Elkin, 2006; Steyerberg *et al*., 2010)—do not prescribe binary outputs. We translated their principles into a structured visual summary to aid the reader. We accept that this format carries an implied precision that the underlying data do not always support, and we acknowledge this as a limitation.

Third, the meta-analysis data in Table 3: Majzoub and Humaidan (2026) raise an important methodological concern: that sensitivity and specificity values derived from Vitthala *et al*. (2026b) may reflect prediction of failure to achieve pregnancy rather than prediction of pregnancy itself, depending on how true positives were defined. This is a valid observation. The true positive definition in Vitthala *et al*. (2026b) followed the conventions of the included studies, in which high SDF in men whose partners did not achieve pregnancy constituted the reference standard. We accept that this framing may not be universally accepted, and that pooling across heterogeneous ART and natural conception settings limits generalizability. This point should have been stated more explicitly in the Opinion article (Vitthala *et al*., 2026a), and we appreciate Majzoub and Humaidan (2026) highlighting it.

Fourth, and most importantly, selective versus routine use: Majzoub and Humaidan (2026) argue that we conflate the absence of evidence for universal use with evidence against selective application. We must be clear: our Opinion article (Vitthala *et al*., 2026a) does not argue against indication-driven SDF testing. We explicitly state that selective use in unexplained infertility, recurrent implantation failure, and recurrent pregnancy loss may provide adjunctive risk stratification. This aligns with European Association of Urology (EAU), ESHRE, and American Urological Association/American Society for Reproductive Medicine (AUA/ASRM) guidelines, all of which support SDF testing in specific scenarios while stopping short of recommending routine use (Esteves *et al*., 2021; Schlegel *et al*., 2021). The ESHRE 2023 guideline on unexplained infertility further acknowledges the lack of standardized thresholds and non-interchangeability of assays as barriers to broader implementation (ESHRE Guideline Group on Unexplained Infertility *et al*., 2023).

Our conclusion that SDF testing should not be incorporated as a universal investigation remains our considered position, consistent with current guidelines. Where we fully agree with Majzoub and Humaidan (2026) is on future research priorities: adequately powered prospective trials must define which patients benefit most from SDF-guided management and whether such strategies improve live birth rates. Until that evidence exists, clinical enthusiasm for SDF testing continues to outpace the data.

We are grateful to Majzoub and Humaidan (2026) for initiating this exchange. Scientific debate of this kind is how the field moves forward.
